# Curcumin as a Perspective Protection for Retinal Pigment Epithelium during Autophagy Inhibition in the Course of Retinal Degeneration

**DOI:** 10.2174/1570159X21666230705103839

**Published:** 2023-09-01

**Authors:** Roberto Pinelli, Michela Ferrucci, Francesca Biagioni, Violet Bumah, Elena Scaffidi, Stefano Puglisi-Allegra, Francesco Fornai

**Affiliations:** 1 SERI, Switzerland Eye Research Institute, Lugano, Switzerland;; 2 Department of Translational Research and New Technologies in Medicine and Surgery, Human Anatomy, University of Pisa, Pisa, Italy;; 3 IRCCS Neuromed, Pozzilli (IS), Italy;; 4 Department of Chemistry and Biochemistry, College of Sciences, San Diego State University, San Diego, CA, U.S.A;; 5 Department of Chemistry and Physics, University of Tennessee, St. Martin, TN, USA

**Keywords:** Phytochemicals, retinal pigment epithelium, autophagy, neurodegeneration, retinal degeneration, 3-methyladenine, age-related macular degeneration

## Abstract

Defective autophagy in the retinal pigment epithelium (RPE) is involved in retinal degeneration, mostly in the course of age-related macular degeneration (AMD), which is an increasingly prevalent retinal disorder, eventually leading to blindness. However, most autophagy activators own serious adverse effects when administered systemically. Curcumin is a phytochemical, which induces autophagy with a wide dose-response curve, which brings minimal side effects. Recent studies indicating defective autophagy in AMD were analyzed. Accordingly, in this perspective, we discuss and provide some evidence about the protective effects of curcumin in preventing RPE cell damage induced by the autophagy inhibitor 3-methyladenine (3-MA). Cells from human RPE were administered the autophagy inhibitor 3-MA. The cell damage induced by 3-MA was assessed at light microscopy by hematoxylin & eosin, Fluoro Jade-B, and ZO1 immunohistochemistry along with electron microscopy. The autophagy inhibitor 3-MA produces cell loss and cell degeneration of RPE cells. These effects are counteracted dose-dependently by curcumin. In line with the hypothesis that the autophagy machinery is key in sustaining the integrity of the RPE, here we provide evidence that the powerful autophagy inhibitor 3-MA produces dose-dependently cell loss and cell degeneration in cultured RPE cells, while inhibiting autophagy as shown by LC3-II/LC3-I ratio and gold-standard assessment of autophagy through LC3-positive autophagy vacuoles. These effects are prevented dose-dependently by curcumin, which activates autophagy. These data shed the perspective of validating the role of phytochemicals as safe autophagy activators to treat AMD.

## 1 INTRODUCTION

The autophagy machinery occurs ubiquitously within eukaryotic cells, where it exerts a number of effects, which are roughly summarized by clearing altered or aged organelles and proteins [1]. Indeed, lipids and sugars are degraded as well, along with additional substrates which are increasingly identified as targets of the autophagy machinery [2]. In specific cells, such an activity is crucial due to inherent needs, which are coped by specific cell types. This is the case of liver cells and neurons. In the retina the removal and prompt turn-over of proteins and organelles are very active at the level of the retinal pigment epithelium (RPE), where autophagy machinery plays a key role in maintaining the physiology of the cells and counteracting an excess of metabolic substrates and damaged organelles [3, 4]. This explains why a defect in the autophagy machinery is now emerging as a converging mechanism to produce retinal degeneration, with a special emphasis on age-related macular degeneration (AMD) [5]. In detail, RPE undergoes early structural alterations in the onset of AMD, which may depend on altered autophagy machinery [5]. Thus, it is not surprising that the pathological hallmark of AMD is represented by extracellular aggregates of undigested, autophagy-dependent substrates known as drusen, which contain cell debris, lipofuscin, altered proteins, glycoproteins, lipids and advanced glycation end products [6, 7]. An early defect which develops between RPE cells consists of the loss of intercellular tight junctions stained for the protein *Zonulae occludentes* 1 (ZO1), which produces a loss of integrity of the barrier between RPE and choroid. This also explains why drusen typically aggregate below the RPE, enlarging the extracellular space between RPE and choroid Bruch’s membrane. For such a reason, very recent studies suggest that improving autophagy in the RPE may ameliorate the course of AMD, which can be induced in specific animal models [8]. Similarly, a significant effect of autophagy modulation is increasingly recognized in a variety of retinal degenerative disorders [9]. In summary, defective autophagy machinery is mostly sensitive in the retina, where the need to turn over and clear various substrates is required. This is in line with the role of a defective autophagy within RPE cells to trigger the onset of AMD and maturate drusen formation [10]. This concept is leading to alternative pathophysiology-rooted strategies in order to find an effective cure for AMD [11]. In keeping with a defective autophagy machinery, a very recent study hypothesized local delivery of the gold-standard autophagy activator rapamycin by lipoprotein nanoparticles [12]. This in-progress strategy is based on the rationale that, rapamycin when administered *in vitro* to RPE cells, clears lipids and lipofuscins which are present within drusen. Such a therapeutic approach, which needs to be validated, is based on the fact that lipoproteins can reach the retina when administered to rats *in vivo*. In fact, chronic systemic administration of rapamycin leads to unbearable side effects for such a long disease course. The present contribution provides a novel perspective, which may lead to an effective therapy by administering a safe natural autophagy activator. Here preliminary data are provided on the strong protective effects induced by the phytochemical curcumin on the expression of ZO1 and survival of RPE cells when the autophagy machinery is severely suppressed occurring in AMD.

## 2 RESULTS AND DISCUSSION

This perspective article reports that curcumin, a powerful and safe autophagy inducer, fully prevents the cell death dose-dependently, which occurs in human RPE cells upon exposure to various doses of the autophagy inhibitor, 3-methyladenine (3-MA). Curcumin administration is also effective in counteracting the loss of ZO1, which is needed to connect the monolayer of RPE cells. Differing from rapamycin, curcumin can be administered systemically for prolonged time intervals. When compared with rapamycin, curcumin exerts a similar amount of neuroprotection. In fact, as reported in Fig. (**1**), curcumin at the dose of 10 μM, fully prevents cell loss and pathological changes induced by the powerful autophagy inhibitor 3-MA administered at moderate (10 mM) and high (20 mM) doses. These effects are reported in Fig. (**1**) and Table **1**. The protective effects of curcumin on the detrimental alterations produced by 3-MA are concomitant with the rescue of autophagy within RPE cells.

**Fig. (2):**
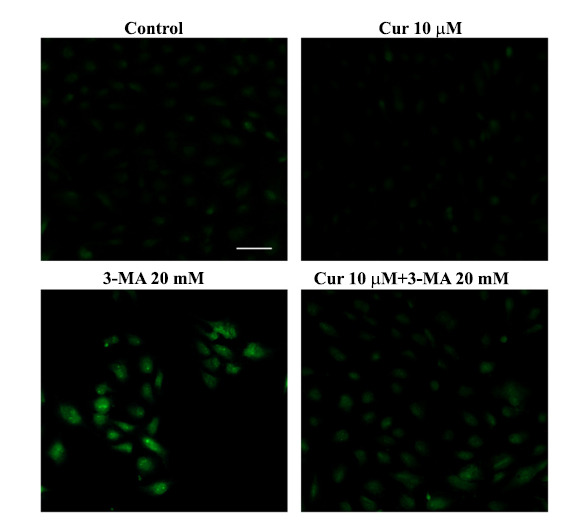
Curcumin prevents the increase in Fluoro Jade-B fluorescence induced by 3-methyladenine. Representative Fluoro Jade-B (FJB) pictures of RPE cells from a control cell culture (Control) and cultures treated with curcumin (Cur, 10 μM) and 3-methyladenine (3-MA, 20 mM), either alone or in combination. RPE cell treatments were carried out as previously described (Fig. **1** legend). FJB staining was carried out in 4% paraformaldehyde fixed RPE cells, which were incubated with 0.06% potassium permanganate for 10 min and then, after washing with distilled water, with 0.0004% FJB solution (consisting of 0.01% FJB in acetic acid) for 20 min. Finally, RPE cells were cover slipped with mounting medium and analyzed at Nikon Eclipse 80i light microscopy, equipped with a fluorescent lamp and a digital camera connected to the NIS Elements software for image analysis. Scale bar = 115 μm.

Similarly, when the fluorescent dye to stain neurodegeneration was used, the marked fluorescence induced by degenerating RPE cells under the effects of 3-MA was occluded by curcumin administration (Fig. **2** and Table **1**).

**Fig. (3):**
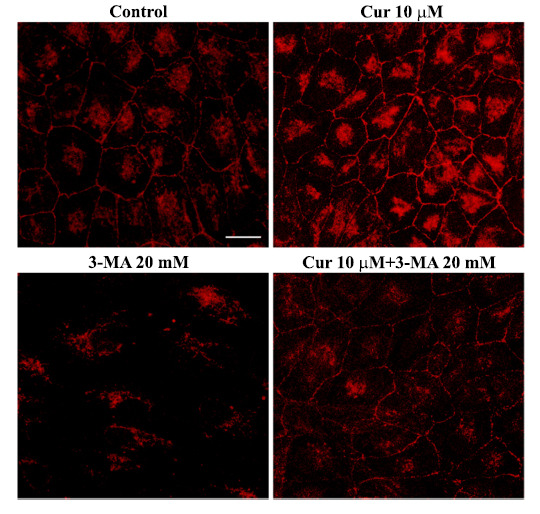
Curcumin prevents the loss of ZO1 immunofluorescence induced by 3-methyladenine. Representative pictures of ZO1 immunofluorescence in RPE cells. ZO1 staining is markedly reduced after 3-MA treatment, while curcumin rescues both the staining and the placement of ZO1 between the RPE cells. RPE cells were treated as previously described (Fig. **1** legend). At the end of the treatments, cells were washed in PBS and fixed with 4% paraformaldehyde in PBS for 15 min, incubated in 0.1% TritonX-100 for 15 min in PBS and then blocked in PBS containing 10% normal goat serum for 1h at room temperature. Cells were then incubated overnight at 4°C in 1% normal goat serum in PBS containing the anti-ZO1 primary antibodies diluted 1:100. After rinsing in PBS, RPE cells were incubated at 21°C for 1 h with the red fluorophore-conjugated secondary antibodies diluted 1:200. Cells were washed in PBS, transferred on coverslip, mounted with the medium Fluoroshield and finally observed under the Nikon Eclipse 80i light microscope equipped with a fluorescent lamp and a digital camera connected to the NIS Elements Software for image analysis. Negative control cells were incubated with secondary antibodies only. Scale bar = 22 μm.

Zonulae occludentes 1 (ZO-1) is a scaffold protein generally used as a gold standard marker to assess the structural and functional integrity of tight junctions between RPE cells [13, 14]. Remarkably, loss of ZO1 is typical in retinal degeneration, mostly in AMD [14-16]. Following the administration of the autophagy inhibitor 3-MA, we reproduced the loss of amount and site specificity of ZO1 between cells (Fig. **3**). This effect is counteracted by curcumin (Fig. **3**).

**Fig. (4):**
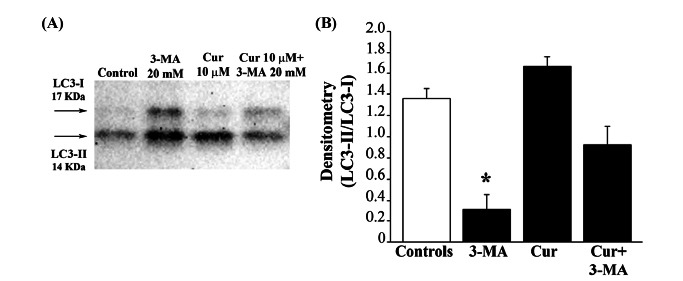
Curcumin counteracts the decrease in LC3-II/LC3-I ratio induced by 3-methyladenine. (**A**) Representative immunoblotting of LC3-II and LC3-I in RPE cells. RPE cells were treated as previously described (Fig. **1** legend). At the end of the treatment, cells were washed out and lysed in a buffer containing protease and phosphatase inhibitors. Then cells were centrifuged at 15,000× g for 20 min at 4°C, the supernatant was collected, and protein concentration was determined using a protein assay kit. Samples containing 40 µg of total proteins were solubilized and electrophoretically resolved using a 12% sodium dodecyl sulphate-(SDS-) polyacrylamide gel and then electro-transferred onto PVDF membranes. Membranes were immersed in a blocking solution with 3% non-fat dried milk in PBS containing 0.1% Tween-20 (TBST) and then were incubated overnight at 4°C with the anti-LC3-I and LC3-II primary antibodies, followed by the appropriate horseradish peroxidase (HRP)-conjugated secondary antibody. The bands were visualized with enhanced chemiluminescence reagents. Image analysis was carried out by ChemiDoc System. (**B**) The graph reports the densitometry of LC3-II/LC3-I ratio, which was performed with ImageJ software. Data were given as mean ± S.E.M. from N = 4 samples per experimental group. *P ≤ 0.05 compared with all other groups.

**Fig. (5):**
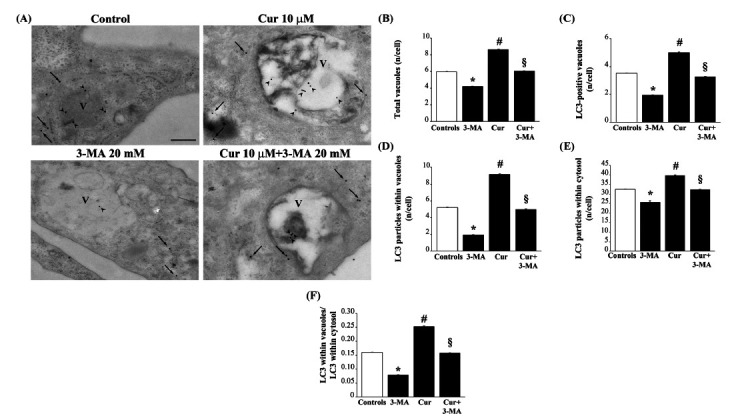
Curcumin counteracts the decrease in LC3-positive autophagy vacuoles induced by 3-methyladenine. (**A**) Representative micrographs of LC3 immunogold particles in RPE cells (arrows) show that 3-MA reduces, while curcumin increases, the localization of LC3 particles within autophagy vacuoles (arrowheads). RPE cells were treated as previously described (Fig. **1** legend). At the end of the treatments, cells were washed in PBS and fixed with 2% paraformaldehyde and 0.1% glutaraldehyde for 90 min at 4°C. After washing in PBS, cells were centrifuged and cell pellet was post-fixed in 1% OsO4 for 1 h at 4°C. Then, cells were dehydrated in ethanol solutions and embedded in epoxy resin. Post-embedding procedure was carried out on ultrathin sections collected on nichel grids. Briefly, grids were incubated in a blocking solution (10% goat serum and 0.2% saponin) for 20 min in a humidified chamber, followed by the primary antibody solution containing the rabbit anti-LC3 primary antibody, diluted 1:50. After washing in PBS, grids were incubated with the secondary antibodies conjugated with gold particles diluted 1:30 in PBS containing 0.2% saponin and 1% goat serum for 1 h. Negative control sections were incubated with the secondary antibody only. Graphs report the ultrastructural stoichiometry of LC3 particles as follows: (**B**) the total number of vacuoles; (**C**) the total number of LC3-positive vacuoles; (**D**) the number of anti-LC3 immunogold particles within vacuoles; (**E**) the number of anti-LC3 immunogold particles within cytosol; (**F**) the number of LC3 immunogold particles within vacuoles out of the number of cytoplasmic LC3 immunogold particles. Data were given as the mean ± SEM per cell counted in 30 cells for each experimental group. *P ≤ 0.05 compared with all other groups; ^#^*P* ≤ 0.05 compared with controls and 3-MA; ^§^*P* ≤ 0.05 compared with 3-MA. V = autophagy vacuole. Scale bar = 230 nm.

This protective effect of curcumin is concomitant to autophagy stimulation, as shown by increased LC3-II/LC3-I ratio (Fig. **4**) and gold standard TEM-aided assessment of autophagy (Fig. **5**).

Materials and Methods used in the present study are reported in detail in the Supplementary material.

The powerful protective effects produced by curcumin on RPE cells to counteract autophagy suppression provide a novel perspective to test curcumin as a safe autophagy activator, which could be administered orally without any device or time-consuming procedure. This approach would avoid the uncertainty of reaching the final target, which may develop over time when multiple ocular applications are required for time intervals lasting several years. This perspective article provides preliminary data, which indicates a powerful protection by curcumin on RPE cells, when autophagy is occluded. Since analogous autophagy defects develop at the onset as well as in the course of AMD, curcumin may represent a novel therapeutic strategy in this degenerative ocular disorder. Such a hypothesis is now being tested in a number of studies. In fact, defective autophagy is present in AMD as recently reported in the comprehensive manuscript by Kaarniranta and co-authors (2022) [5], who found that in AMD a defect in the autophagy pathway drives a number of pathological events such as the occurrence of damage and atrophy of the RPE featuring stagnant lysosome and lipofuscin aggregates, which may be released in the extracellular space to form drusen along with protein misfolding and protein glycation. Additionally, defective autophagy brings to uncontrolled inflammatory response, which may worsen AMD leading to retinal geographic atrophy and fibrosis as well as neovascularization. The present perspective may be retrospectively considered when interpreting recent data. In fact, recent evidence indicates that phytochemicals produce beneficial effects or even reverse the visual deterioration and drusen accumulation in patients suffering from AMD [3, 4, 17-19]. In this scenario, the full protective effects of curcumin in AMD are likely to depend on the strong stimulation of autophagy activity exerted by curcumin [20]. This effect is validated in human RPE, where curcumin is shown here to rescue the autophagy flux following autophagy inhibition, as previously reported [21, 22].

## CONCLUSION

Autophagy inhibition is emerging as a pathogenic mechanism in the course of retinal degeneration [23-25]. This is most evident at the onset and during the progression of AMD [26-29]. Most autophagy inducers own serious side effects. Recent evidence indicates that specific phytochemicals may act as powerful protective agents by promoting autophagy. Curcumin is a powerful natural compound which counteracts autophagy inhibition. The damage to RPE following exposure to the autophagy inhibitor 3-MA is reversed dose-dependently by the administration of curcumin. These protective effects call for extensive studies aimed to challenge natural phytochemicals as potential protective agents in retinal disorders featuring a defective autophagy pathway. At the same time, novel studies are required to dissect which specific steps are affected in the autophagy machinery when AMD starts and develops.

## Figures and Tables

**Fig. (1):**
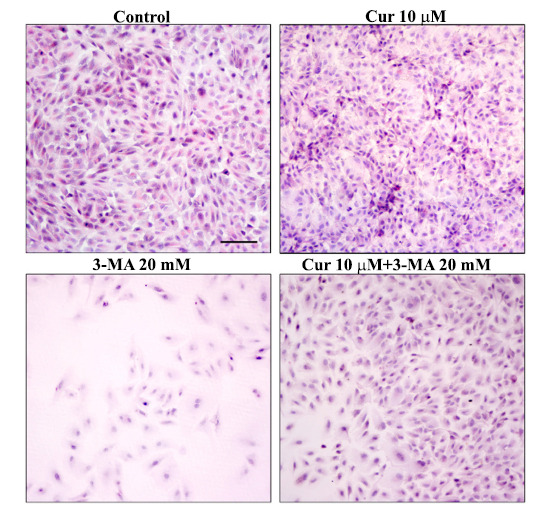
Curcumin protects RPE cells against cell loss induced by 3-methyladenine. Representative Hematoxylin & Eosin (H&E) pictures of RPE from a control cell culture (Control) and cultures treated with curcumin (Cur, 10 μM) and 3-methyladenine (3-MA, 20 mM), either alone or in combination. RPE cells elevate retina pigment epithelia (ARPE 19 cell line). RPE cell treatments were carried out as follows. A stock solution of curcumin 9.5 mM was prepared by dissolving 3.5 mg of curcumin powder in 1 mL of DMSO. Final concentrations of curcumin (1 μM and 10 μM) were obtained by diluting aliquots of the stock solution within the culture medium. 3-Methyladenine was dissolved in the culture medium at 10 mM and 20 mM concentrations. In single treatment experiments, RPE was exposed to curcumin or 3-methyladenine for 72 h. In combined (Cur+3-MA) experiments, curcumin was added to RPE cell culture 2 hours before 3-MA. H&E staining was carried out on RPE cells fixed with 4% paraformaldehyde for 15 min. In detail, first RPE was stained with hematoxylin for several minutes and then the cells were stained with eosin solution. After repeated washing with distilled water to remove the excess dye, cells were dehydrated in increasing alcohol solutions, clarified in xylene, and finally covered with DPX mounting medium. Stained cells were observed under Nikon Eclipse 80i light microscope, equipped with a digital camera connected to the NIS Elements software for image analysis. Scale bar = 115 μm.

**Table 1:** Protective effects of curcumin (Cur) against RPE damage induced by the autophagy inhibitor 3-methyladenine (3-MA).

**Treatment**	**H&E-positive Cells (% of Controls)**	**FJB-positive Cells (Number of Cells)**
Controls	100.00 ± 4.45	26.67 ± 2.87
Cur 1 mM	99.45 ± 3.10	27.22 ± 2.37
Cur 10 mM	98.29 ± 7.46	28.89 ± 2.36
3-MA 10 Mm	32.25 ± 2.92*	121.56 ± 11.65*
3-MA 20 mM	17.48 ± 1.86*	192.89 ± 9.80*
Cur 1 mM+3-MA 10 mM	71.25 ± 3.48^§^	83.33 ± 7.62^§^
Cur 1 mM+3-MA 20 mM	29.04 ± 1.92*	177.67 ± 6.5*
Cur 10 mM+3-MA 10 mM	93.11 ± 4.19^#^	26.89 ± 2.82^#^
Cur 10 mM+3-MA 20 mM	83.57 ± 4.54^^^	30.78 ± 3.41^^^

**Note:** Table reports data related to cell counts which were carried out in H&E or FJB-stained RPE cells. In detail, H&E-stained cells were counted under 10x magnification and values are expressed as the mean percentage ± S.E.M. of cells counted in three independent experiments (assuming control cells as 100%). The number of FJB-positive cells was counted under 10x magnification and values are expressed as the mean number ± S.E.M. of FJB-positive cells from three independent experiments.

*p < 0.05 compared with controls; ^§^*p* < 0.05 compared with 3-MA 10 mM and controls; ^#^*p* < 0.05 compared with 3-MA 10 mM; ^^^*p* < 0.05 compared with 3-MA 20 mM.

## LIST OF ABBREVIATIONS

AMD: Age-related Macular Degeneration
3-MA: 3-Methyladenine
RPE: Retinal Pigment Epithelium
ZO1: Zonulae Occludentes 1
