# Supplementary material for: Curcumin as a Perspective Protection for Retinal Pigment Epithelium during Autophagy Inhibition in the Course of Retinal Degeneration
Source: Curr Neuropharmacol. 2023 Sep 1;21(11):2227–32. doi: 10.2174/1570159X21666230705103839 (PMC10556393; doi:10.2174/1570159X21666230705103839)
Supplement: Supplementary file 1 — Supplementary material is available on the publisher’s website along with the published article. [file CN-21-2227_SD1.pdf]

Supplementary Material

Curcumin as a Perspective Protection for Retinal Pigment Epithelium during Autophagy Inhibition in the Course of Retinal Degeneration

Roberto Pinelli[1,#], Michela Ferrucci[2,#], Francesca Biagioni[3,#], Violet Bumah[4,5], Elena Scaffidi[1], Stefano Puglisi-Allegra[3] and Francesco Fornai[2,3,*]

[1]SERI, Switzerland Eye Research Institute, Lugano, Switzerland; [2]Department of Translational Research and New Technologies in Medicine and Surgery, Human Anatomy, University of Pisa, Pisa, Italy; [3]IRCCS Neuromed, Pozzilli (IS), Italy; [4]Department of Chemistry and Biochemistry, College of Sciences, San Diego State University, San Diego, CA, U.S.A; [5]Department of Chemistry and Physics, University of Tennessee, St. Martin, TN, USA

SUPPLEMENTARY MATERIALS AND METHODS

Cell Cultures and Treatments

Experiments were carried out in human RPE cells, which arise human retina pigment epithelia (ARPE 19 cell line). ARPE-19 cell culture was purchased from the IPSLER Institute (Istituto Zooprofilattico Sperimentale della Lombardia e dell'Emilia Romagna, Brescia, Italy). Cells were cultured in Dulbecco's Modified Eagle Medium mixed with Ham's F-12 medium (DMEM/F12 40% respectively; Sigma-Aldrich, St Louis, MO, USA), supplemented with 20% of fetal bovine serum (FBS; Sigma-Aldrich) and penicillin-streptomycin (100 IU/mL-100 mg/mL; Sigma-Aldrich). Cells were cultured at 37°C in a humidified incubator containing 5% CO_2. ARPE cells were used for experiments after they reached approximately 80% confluence. At this point of their growth, the cells were digested with 0.25% trypsin, harvested and seeded in multi-well plates for experiments.

Timing and dosing of 3-methyladenine (3-MA) and curcumin (Cur) treatments were selected based on pilot experiments and previous studies [1], in order to obtain a frank inhibition and activation of autophagy, respectively. In detail, cell treatments were carried out as follows. A stock solution of Cur (Sigma-Aldrich) 9.5 mM was prepared by dissolving 3.5 mg of Cur powder in 1 mL of dimethyl sulphoxide (DMSO, (Sigma-Aldrich). Final concentrations of Cur (1 μM and 10 μM) were obtained by diluting aliquots of the stock solution within the culture medium. 3-Methyladenine (Sigma-Aldrich) was dissolved in the culture medium at the concentrations of 10 mM and 20 mM. In combined (Cur+3-MA) experiments, Cur was added to RPE cell culture 2 hours before 3-MA. RPE were exposed to either single or combined treatments for 72 h.

Hematoxylin and Eosin (H&E) histochemistry

For H&E staining, 5×10^4 ARPE cells were seeded on poly-lysine slides and placed in 24-well plates containing 1 mL/well of culture medium. At the end of treatments, cells were fixed using 4% paraformaldehyde in phosphate-buffered saline (PBS) solution for 15 min, washed in PBS and immersed for some minutes in hematoxylin solution (Sigma-Aldrich). Then, the slides were washed out to stop hematoxylin staining and immediately plunged within the eosin solution (Sigma-Aldrich). After repeated washing to remove the excess of dye, cells were dehydrated using increasing alcohol solutions (from 70% to 100% alcohol concentration) and clarified in xylene. Slides were transferred on coverslips, covered with DPX mounting medium (Sigma-Aldrich), and finally observed under a Nikon Eclipse 80i light microscope (Nikon, Tokyo, Japan). Cell count was performed at light microscopy at 20× magnification; the number of H&E-stained cells detectable after each specific treatment was counted and expressed as the mean percentage ± SEM of the control group (assuming Controls as 100%). Data were obtained from three independent experiments.

1875-6190/23 $65.00+.00 © 2023 Bentham Science Publishers

Fluoro Jade B (FJB) histofluorescence

Fluoro Jade B (FJB) staining [2] was carried out in 5×10^4 ARPE cells, which were grown on poly-lysine slides placed in 24-well plates, in a final volume of 1 mL/well. At the end of the treatments cells were washed in PBS, fixed with paraformaldehyde 4% for 5 min, and incubated with 0.06% potassium permanganate for 10 min at room temperature. After washing in distilled water, cells were incubated with 0.0004% FJB (Merck Millipore, Billerica, MA, USA) solution, consisting in 0.01% FJB in acetic acid, at room temperature for 20 min. Then, slides were transferred on coverslips and covered with the mounting medium DPX (Sigma-Aldrich). FJB-positive cells were analyzed at Nikon Eclipse 80i light microscopy (Nikon, Tokyo, Japan), equipped with a fluorescence lamp and a digital camera connected to the NIS Elements software for image analysis (Nikon, Tokyo, Japan). For each experimental group, the count of FJB-positive cells was carried out at 20× magnification and values were expressed as the mean number±SEM counted in each experimental group. All data were obtained from three independent experiments.

Immunohistochemistry

ARPE cells ($N=5\times10^4$) were grown on poly-lysine slides and placed in 24-well plates containing 1 mL/well of culture medium. At the end of the treatments, cells were washed in PBS and fixed with 4% paraformaldehyde in PBS for 15 min. Fixed cells were incubated in 0.1% Tri-tonX-100 for 15 min in PBS, and blocked in PBS+10% normal goat serum for 1h at room temperature. Cells were then incubated overnight at 4°C in 1% normal goat serum in PBS containing the anti-ZO1 *(zonulae occludentes* 1) primary antibodies (Sigma-Aldrich) diluted 1:100. After washing in PBS, ARPE cells were incubated with the appropriate red fluorophore-conjugated secondary antibodies Alexa 594 (Life Technologies, Carlsbad, CA, USA) diluted 1:200 for 1h at room temperature. Then, cells were washed in PBS, transferred on coverslip and they were mounted with the mounting medium Fluoroshield (Sigma-Aldrich). Finally, ARPE cells cells were observed under the Nikon Eclipse 80i light microscope (Nikon) equipped with a fluorescent lamp and a digital camera connected to the NIS Elements Software for image analysis (Nikon). Negative control cells were incubated with secondary antibodies only.

Transmission Electron Microscopy (TEM)

ARPE cells (1×10^6) were seeded in 10 mm diameter culture dishes with 5 mL of culture medium. After removing culture medium, cells were fixed with a fixing solution containing 2.0% paraformaldehyde and 0.1% glutaraldehyde in 0.1 M PBS (pH 7.4) for 90 min at 4° C. This aldehyde concentration minimally covers antigen epitopes, while fairly preserving tissue architecture. After removal of the fixing solution, cells were gently scraped from the plate, centrifuged at 10,000 rpm for 10 min and cell pellet was collected, washed in PBS, and post-fixed in 1% OsO4 for 1 h at 4° C. Then, cell pellet was washed in PBS, dehydrated in increasing ethanol solutions and finally embedded in epoxy resin.

Ultra-thin sections were obtained at ultra-microtome (Leica Microsystems, Wetzlar, Germany), were stained with uranyl acetate and lead citrate, and they were finally examined using a JEOL JEM-100SX transmission electron microscope (JEOL, Tokyo, Japan).

Post-embedding immuno-electronmicroscopy

Post-embedding procedure was carried out on ultrathin sections collected on nickel grids, which were incubated on droplets of aqueous sodium metaperiodate (NaIO4), for 30 min, at room temperature. $NaIO_4$ is an oxidizing agent which allows a closer contact between antibodies and antigens by removing OsO_4 [3]. This step improves the visualization of immunogold particles within a sharp context of cell integrity, thus allowing the counting of molecules within specific cell compartments. Grids were washed in PBS and incubated in a blocking solution containing 10% goat serum and 0.2% saponin for 20 min in a humidified chamber, at room temperature. Then, grids were incubated with the primary antibody solution containing the rabbit anti-LC3 (Abcam, Cambridge, UK) primary antibody, diluted 1:50. After washing in PBS, grids were incubated with the secondary antibodies conjugated with gold particles of 20 nm mean diameter (BB International), diluted 1:30 in PBS containing 0.2% saponin and 1% goat serum for 1 h, at room temperature. Negative control sections were incubated with the secondary antibody only. After washing in PBS, grids were incubated on droplets of 1% glutaraldehyde for 3 min.

Ultrastructural morphometry

Ultrastructural morphometry of autophagy vacuoles and anti-LC3 immunogold was performed directly at TEM at a magnification of 8000x [4] since this represents the minimal magnification at which immunogold particles and all cell organelles can be concomitantly identified. Briefly, we started to count from a grid square corner in order to scan the whole cell pellet within that grid square, which was randomly identified. According to Lenzi *et al.* [5], we counted the number of unstained vacuoles per cell as vacuoles with single, double, or multiple membranes possessing the same electron density of the surrounding cytoplasm or containing some electron dense structure. In each cell, we counted: (i) the total number of vacuoles; (ii) the total number of LC3-positive vacuoles; (iii) the number of anti-LC3 immunogold particles within vacuoles; (iv) the number of anti-LC3 immunogold particles in the cytoplasm; (v) the number of LC3 immunogold particles within vacuoles out of the number of cytoplasmic LC3 immunogold particles. We expressed these data as the mean±SEM per cell. Several grids were analyzed in order to count a total number of 30 cells for each experimental group.

Western Blotting

ARPE cells were treated as previously reported for 72h. At the end of the treatments cells were washed out and were lysed in a buffer (100 mM Tris-HCl, pH 7.5, 5 M NaCl, 0.5 m EDTA, 10% SDS, 1% NP40, IGEPAL) containing protease and phosphatase inhibitors. Then cells were centrifuged at 15,000× g for 20 min at 4 °C, the supernatant was collected, and protein concentration was determined using a protein assay kit (Sigma-Aldrich). Samples containing 40 µg of total proteins were solubilized and electrophoretically resolved using a 12% sodium dodecyl sulphate-(SDS-) polyacrylamide gel. Following electrophoresis, proteins were electro-transferred onto PVDF membranes (Bio-Rad Laboratories, Hercules, CA, USA) by a semi-dry system (BioRad Laboratories). Membranes were immersed in a blocking solution with 3% non-fat dried milk in PBS containing 0.1% Tween-20 (TBST) and then were incubated overnight at 4 °C with the anti-LC3-I and LC3-II (MBL International, Woburn, Ma 01801 USA) primary antibodies diluted 1:1000. After extensive washing with TBST, blots were incubated with the appropriate horseradish peroxidase (HRP)-conjugated secondary antibody (Amersham Biosciences, Amersham, UK) diluted 1:3000 for 1 h, at room temperature. The bands were visualized with enhanced chemiluminescence reagents (GE Healthcare Biosciences, Little Chalfont, Buckinghamshire, UK). Image analysis was carried out by ChemiDoc System (Bio-Rad Laboratories). Densitometric analysis of LC3-II/LC3-I ratio was performed with ImageJ software (NIH, Bethesda, MD, USA, Version 1.8.0_172). The intensity of the blotting was expressed as the mean±S.E.M. of the optical density measured for each experimental group, and obtained from four independent experiments.

Statistics

Data on the amount of cell survival were expressed as the mean percentage±SEM of H&E-positive cells (assuming controls=100%) from three independent experiments. Data on the amount of degenerating cells were expressed as the mean±SEM of FJB-positive cells counted in each experimental group and obtained from three independent experiments.

For ultrastructural morphometry data were given as an absolute number concerning the following measurements: (i) unstained total vacuoles per cell (ii) the total number of LC3-positive vacuoles; (iii) the number of anti-LC3 immunogold particles within vacuoles; (iv) the number of anti-LC3 immunogold particles in the cytoplasm; (v) the number of LC3 immunogold particles within vacuoles out of the number of cytoplasmic LC3 immunogold particles. All data were reported as the mean±SEM per cell from 30 cells per group.

For Western blot optical density was expressed as the mean±SEM calculated in N=4 samples per group.

All statistical analyses were carried out by using one-way analysis of variance, ANOVA, followed by Sheffè's *post hoc* analysis. Null hypothesis (H_0) was rejected for $P \leq 0.05$.

REFERENCES

[1] Ryskalin L, Puglisi-Allegra S, Lazzeri G, Biagioni F, Busceti CL, Balestrini L, Fornasiero A, Leone S, Pompili E, Ferrucci M, Fornai F. Neuroprotective Effects of Curcumin in Methamphetamine-Induced Toxicity. Molecules. 2021 Apr 24;26(9):2493. doi: 10.3390/molecules26092493.

[2] Schmued LC. Hopkins KJ. Fluoro-Jade B: A high affinity fluorescent marker for the localization of neuronal degeneration. 2000 Aug 25;874(2):123-130. doi: 10.1016/s0006-8993(00)02513-0.

[3] Bendayan M, Zollinger M. Ultrastructural localization of antigenic sites on osmium-fixed tissues applying the protein A-gold technique. J Histochem Cytochem. 1983 Jan;31(1):101-109. doi: 10.1177/31.1.6187796.

[4] Lucocq M, Habermann A, Watt S, Backer JM, Mayhew TM, Griffiths G. A rapid method for assessing the distribution of gold labeling on thin sections. J Histochem Cytochem. 2004 Aug;52(8):991-1000. doi: 10.1369/jhc.3A6178.2004.

[5] Lenzi P, Lazzeri G, Biagioni F, Busceti CL, Gambardella S, Salvetti A, Fornai F. The Autophagoproteasome a Novel Cell Clearing Organelle in Baseline and Stimulated Conditions. Front Neuroanat. 2016 Jul 21;10:78. doi: 10.3389/fnana.2016.00078.
